# Sensing marine biomolecules: smell, taste, and the evolutionary transition from aquatic to terrestrial life

**DOI:** 10.3389/fchem.2014.00092

**Published:** 2014-10-16

**Authors:** Ernesto Mollo, Angelo Fontana, Vassilios Roussis, Gianluca Polese, Pietro Amodeo, Michael T. Ghiselin

**Affiliations:** ^1^Institute of Biomolecular Chemistry, National Research Council of ItalyPozzuoli, Italy; ^2^Department of Pharmacy, University of AthensAthens, Greece; ^3^Department of Biology, University of Naples “Federico II,”Naples, Italy; ^4^Department of Invertebrate Zoology and Geology, California Academy of SciencesSan Francisco, CA, USA

**Keywords:** marine natural products, terpenoids, olfaction, gustation, volatility, solubility, odorant receptors, GPCRs

## Abstract

The usual definition of smell and taste as distance and contact forms of chemoreception, respectively, has resulted in the belief that, during the shift from aquatic to terrestrial life, odorant receptors (ORs) were selected mainly to recognize airborne hydrophobic ligands, instead of the hydrophilic molecules involved in marine remote-sensing. This post-adaptive evolutionary scenario, however, neglects the fact that marine organisms 1) produce and detect a wide range of small hydrophobic and volatile molecules, especially terpenoids, and 2) contain genes coding for ORs that are able to bind those compounds. These apparent anomalies can be resolved by adopting an alternative, pre-adaptive scenario. Before becoming airborne on land, small molecules, almost insoluble in water, already played a key role in aquatic communication, but acting in “contact” forms of olfaction that did not require major molecular innovations to become effective at a distance in air. Rather, when air was “invaded” by volatile marine terpenoids, an expansion of the spatial range of olfaction was an incidental consequence rather than an adaptation.

Olfaction (the sense of smell) is generally defined as the ability of terrestrial organisms to detect volatile molecules coming from a distance in the air, whereas in aquatic habitats waterborne signaling is considered the counterpart of airborne signaling. This definition is based on criteria that are “spatial” (the distance between the emitter and the receiver of the signal) rather than “molecular” (interactions between ligands and receptors). The different criteria, however, are not in conflict with each other when only organisms living in the aerial medium are considered. It is widely accepted that the first step in odor perception takes place when odorant airborne molecules—generally compounds with a molecular weight (MW) smaller than ~300 Da (Mori et al., 2006; Touhara and Vosshall, 2009)—are transported by air, and finally bind to specific sites on odorant receptors (ORs) expressed in olfactory sensory neurons that transmit signals to the brain (Buck, 2000). Difficulties with the above definition of olfaction based on signal range emerge, however, when considering aquatic environments, where solubility, instead of volatility, is the crucial necessary condition for the long-distance transport of biomolecules. Many marine organisms, in fact, have a strong smell but only if they are taken out of the water, because their odorant molecules are hydrophobic and therefore cannot be effective in any form of remote sensing based on diffusion in water. They are mainly small representatives of the largest class of natural products, the terpenoids (isoprenoids), which are widespread both in marine and terrestrial organisms. Although it may seem curious, these marine metabolites (Figure 1, yellow spots) should be included in the group of biogenic volatile organic compounds (BVOCs) acting as mediators of growth, development, reproduction and, especially, defense, of many land plants and animals. Remarkably, some of the volatile terpenoids that have been found in marine sponges and nudibranchs (e.g., the odorant furanosesquiterpenes longifolin and dendrolasin) have also been found in terrestrial plants and insects (Pietra, 1995). As a further example, but with a special interest in pharmacology, the strong-smelling liposoluble terpenoid furanodiene showing important pharmacological properties, including anti-cancer activity (Dolara et al., 1996; Ba et al., 2009; Zhong et al., 2012a,b,c; Buccioni et al., 2014; Xu et al., 2014), is a component of both terrestrial plants and marine benthic invertebrates (Bowden et al., 1980; McPhail et al., 2001; Gavagnin et al., 2003). Therefore, given that olfaction (the sense of smell) is generally regarded as a distance sense, while gustation (the sense of taste) is a contact sense (Smith, 2008), exactly the same volatile molecules, almost insoluble in water, would be considered at the same time as being smelled on land, and tasted by contact at sea. Strangely enough, it has been emphasized that the “gustatory” perceptions of terrestrial tetrapod vertebrates, and the “olfactory” perceptions of fish provided with a sense of smell, are both mediated by stimulating molecules in solution (Smith, 2008). The above incongruities are certainly among the main reasons why evolutionary biologists have not yet been able to write a satisfactory historical narrative on chemoreception, which should consistently relate sequences of contingent historical events to laws of nature (Ghiselin, 1997; Cimino and Ghiselin, 2001).

**Figure 1:**
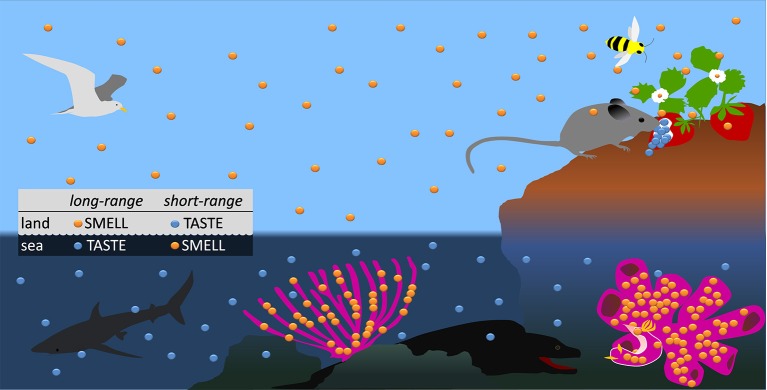
**Schematic distribution of airborne/hydrophobic (yellow spots) and non-volatile/waterborne (blue spots) biomolecules in terrestrial and marine environments**. The box summarizes the range of the chemical senses in the different environments, when mediated by the above chemical cues.

Particularly unpersuasive is the current representation of what happened during the conquest of the land by aquatic organisms. It has been proposed that a successful transition to terrestrial life should have raised dramatically new demands on the chemosensory system, due to the fact that the olfactory stimuli changed from hydrophilic to mainly hydrophobic and airborne molecules (Krång et al., 2012; Tuchina et al., 2014). On the other hand, the traditional notion that olfaction is a “distance sense” led many authors to believe that under water the sense of smell is mediated almost exclusively by waterborne signaling molecules (Ache and Young, 2005; Eisthen and Polese, 2006; Smith, 2008; Shi and Zhang, 2009; Brönmark and Hansson, 2012; Krång et al., 2012; Tuchina et al., 2014). Accordingly, we should consider odorants to be those compounds that are easily dissolved in water. This would certainly embrace dissolved gasses, peptides, proteins, and functionalized hydrocarbons, which are known to function as chemical signals within pelagic interactions (Pohnert et al., 2007), including the non-volatile highly water soluble osmolyte dimethylsulfoniopropionate (DMSP), the volatile and hydrophobic enzymatic breakdown product of which, dimethyl sulfide (DMS), is emitted to the atmosphere and provides a foraging cue for seabirds (Savoca and Nevitt, 2014). Even though the structural differences between DMSP and DMS suggest that they are detected by different receptors, both compounds are evidently involved in long-range forms of chemical communication, pertaining, by definition, to olfaction. However, one would ask what is the actual meaning of olfactory waterborne remote-signaling (Figure 1, blue spots) in the majority of sessile marine benthic organisms, such as sponges and soft corals. Their interactions with predators or conspecifics occur, in fact, at extremely close range, and are often mediated by lipophilic compounds. Moreover, the chemoreception of chemical signals is evidently the main system for navigation in slow-moving animals such as nudibranch gastropods. According to the current view, therefore, the tactile detection of lipophilic odorants by nudibranchs is “taste,” even when the chemical signals are exactly the same as those “smelled” by land animals.

Biased interpretations of related results in molecular genetics do not help to shed light on the issue. To overcome a sort of molecular “impasse” in chemoreception, it has been proposed that OR genes found in marine organisms encode ORs that detect water-soluble odorants (Niimura and Nei, 2005; Niimura, 2012). Therefore, in spite of our very limited knowledge of the actual specificity of ORs for ligands, subjective categories in molecular genetics have been built up to integrate the phyletic distribution of chemosensory genes, especially in marine organisms, with the traditional definition of the chemical senses based on the distance between the emitter and the detector. Different classes of receptors have been thus hypothesized on the basis of the type of odorous ligands supposedly recognized in the different environments, class I for water soluble, class II for airborne odorants. Moreover, OR genes in teleost fishes and tetrapods have been classified in the seven groups α-η, of which groups α and γ, on the basis of their common occurrence in terrestrial animals, have been assumed to detect airborne odorants (Niimura, 2012). Accordingly, the unexpected presence of α and γ genes in the coelacanth fish *Latimeria chalumnae*, whose genome was recently sequenced (Amemiya et al., 2013), has been explained by the hypothesis that an ancestral coelacanth lineage once inhabited shallow water and then returned to greater depths (Nikaido et al., 2013). But, how could this hypothesis be reasonably extended to explain the finding of an intact functional γ OR gene in zebrafish too (Niimura and Nei, 2005; Picone et al., 2014), for which an “aerial past” has not yet been suggested? However, even leaving aside the evident contradictions originating from the application of this kind of conjecture to totally aquatic species, once again, it would seem, they are evidently based on the unfounded assumption that aquatic fishes can only sense waterborne chemicals by olfaction, whereas terrestrial vertebrates mainly sense volatile airborne chemicals. This is a patently obvious error, given the implausible “sudden” appearance, during the conquest of land by aquatic organisms, of both the volatile compounds, and the extremely complex chemoreceptorial machinery able to bind and decode such chemical signals (Nara et al., 2011). What we have here would seem to be tradition masquerading as facts, and anomaly being explained away by *ad hoc* hypotheses.

ORs are G protein coupled receptors (GPCRs) belonging to the rhodopsin-like gene family, the first representatives of which seem to have appeared between 800 and 580 million years ago, being present in marine cnidarians, placozoans, and sponges (Römpler et al., 2007; Srivastava et al., 2008, 2010; Churcher and Taylor, 2010). In particular, the antiquity of ORs is clear from the presence of their orthologs both in the cnidarian *Nematostella vectensis*, and in the cephalochordate *Branchiostoma floridae* (Churcher and Taylor, 2009, 2010). This demonstrates that ORs evolved at least 550 million years ago in marine invertebrates. On the other hand, marine natural product chemists have found massive evidence of marine organisms communicating by liposoluble secondary metabolites, most of which play critical ecological roles (Cimino and Ghiselin, 2001, 2009; Mollo et al., 2008). In particular, marine animals that are unable to escape predators by rapid locomotion provide some of the best documented examples of defensive strategies based on the use of allomones. Nonpolar terpenoids are commonly released on the body surface of these animals or in their tracks, but can also be accumulated in sacrificial parts of the body, to be locally detected by predators at extremely high concentrations (Carbone et al., 2013). Remarkably, considerable structural variability among the protective compounds, which include both polar and nonpolar metabolites, suggests that solubility in water did not play a critical role in the evolution of chemical defense in marine invertebrates (Pawlik, 2012). Marine liposoluble terpenoids have also been studied for their ability to indicate a food source for the receiver (kairomones), stimulating feeding once prey have been contacted (Hay, 2009), while volatile terpenes have been also found to act as gamete attractants (pheromones) in brown algae and cnidarians (Jaenicke and Boland, 1982; Coll et al., 1995). Notably, the subset with MW <300 Da of water-insoluble molecules that enable many marine benthic invertebrates to repel predators, and reproduce successfully, also impart a characteristic smell to those organisms when exposed to air. According to the current view, however, the aquatic detection of those small molecules, which are both odorants on land and insoluble in water, should be called “gustation,” a sense requiring physical contact with the emitter (Smith, 2008). But, given the premise on the nature of the odorant molecules and their macromolecular counterparts, this argument doesn't make sense; actually it is a *non sequitur*. That the molecules in question must be in contact with the receptors is true of both olfaction and gustation. Volatile terpenoids, however, are among the odorants recognized by ORs on land. How can we accept, then, the idea that both those ligands and the related ligand-receptor complexes lose their sensorial specificity just by moving to the sea? This is unacceptable, unless one changes the name of the receptors when they operate underwater. We should, instead, consider that there is no “action at a distance” in the specific ligand-receptor recognition step.

But there are other arguments against the traditional definition of the chemical senses. Solubility and volatility are affected by local physico-chemical parameters. Thus changes in the local conditions could switch the perception of the same chemical message from “gustative” to “olfactive,” and vice versa. In addition, lipophilic compounds can move long distances in water in the form of micellae, much as nonvolatile compounds can be transported in the atmosphere in the form of aerosol particles.

Overall, we have logical reasons to believe that the persistent habit of giving priority to the detection of distant objects in defining the sense of smell in different environments is not really consistent with the recent discovery of the molecular basis for odor recognition (Buck and Axel, 1991), especially for its evolutionary implications. In fact, it would have required an abrupt, extensive and concerted change in the complex patterns of affinity of the ORs for ligands during the transition from water to land, something that would be highly unlikely given the highly combinatorial character of the molecular mechanisms of olfaction (Nara et al., 2011). Moreover, it generates a contradiction in terms, which, not being merely a semantic problem seems to invoke a simplistic unifying theory for chemoreception where taste and smell lose their distinctive features. In this report we propose, instead, a new perspective aiming at a radical solution to this problem, at the same time preserving the usual taste-smell dichotomy.

From our perspective, the transition from aquatic, to semiaquatic, and terrestrial olfaction certainly required physiological and anatomical adaptations in the chemosensory systems, but did not result in dramatic changes in the complex patterns of affinity of the ORs for ligands. Thus, by assigning a central role to both ligands and receptors involved in this process, and given the evident “molecular continuity” of terpenoid ligands across the transition from aquatic to terrestrial life, we reinterpret the aquatic detection of volatile compounds as the ancient precursor of terrestrial olfaction. Low-solubility in water and volatility in air, common features of the odorant terpenoids, determine opposite communication ranges in the two media, the former enhancing efficacy of short-range or contact communication in water by preventing, or strongly limiting, the dilution of the signal in the medium, the latter allowing long-range communication by dispersion of the signal in air (Figure 1). However, the kinds of messages are preserved in both environments, thus making the present perspective sound from an evolutionary point of view. Within this framework, many other important changes observed in the anatomical features of chemosensory organs of terrestrial animals, and in the cellular, sub-cellular, and molecular mechanisms that mediate sensing and processing of chemical stimuli, have to be rather considered post-adaptive phenomena. They have allowed the detection of odorants at astonishingly low concentrations in air, where the chemical signal is extremely diluted and covers long distances, and, together with other neurophysiological and anatomical adaptations, the correct spatial and temporal resolution of the signals. Examples of these adaptations possibly include the enormous increase of the number of OR genes in the terrestrial tetrapods, relative to marine fish (Niimura, 2012) and the appearance of odorant receptor-coreceptor complexes in insects exhibiting different signal transduction mechanisms, in which G-proteins are only partially or not at all involved (Sato et al., 2008; Wicher et al., 2008).

Unlike that based on signal range, our interpretation of chemoreception can be extended even to very peculiar forms of chemical communication. For instance, at the interface of the aquatic and aerial environments, some marine insects can communicate by detecting pheromones that, being partly hydrophobic and partly hydrophilic, are neither airborne nor waterborne and can disperse in two dimensions on the sea surface, allowing mate location (Tsoukatou et al., 2001). In this case discrimination between smell and taste based of the signal spatial range would certainly be confusing without considering specific molecular interactions of the ligands with chemosensory receptors. This latter approach, however, calls for a rigorous classification of the chemoreceptors and their ligands fully based on molecular and genetic criteria, which will require a better knowledge of both genomes, and interaction patterns. Such studies are certainly desirable both for a better understanding of all forms of chemoreception, and to shed more light on the related anatomical, functional, and physiological adaptations. There can be no doubt, however, that the transition from an aquatic to terrestrial life did not involve dramatic changes in the chemoreception systems, even in those involved in both the perception by contact of non-volatile lipophilic compounds (e.g., terpenoids with MW >300 Da), and the detection at a distance of odorants that are both volatile and waterborne (e.g., amines). In these cases both receptors, and their range, have evidently been conserved. The fact nonetheless remains that the shift from a contact (at sea) to a remote (on land) form of olfaction is logically sound for the detection of the most abundant group of BVOCs (Blanch et al., 2009), namely the small terpenoids detected by ORs.

These considerations also suggest that we ought to reconsider what we call “smell” and “taste” in marine environments, where species live immersed in water, and distance chemoreception of hydrophilic substances is a straightforward matter. Thus, based on molecular criteria, what is currently called “aquatic olfaction” of waterborne molecules should be reasonably considered the real aquatic sense of taste, exploiting molecular mechanisms similar to those involved in terrestrial gustatory perception, where “the stimulating molecules have to be in solution and in contact with the receptor” (Smith, 2008). The rationale is compelling given that neither sugars nor glutamate, for instance, may be regarded as olfactory molecules by the mere fact that they can be perceived in their dissolved form in water, knowing, *inter alia*, that the existence of sweet and umami taste receptors (TRs) specifically binding the mentioned class of compounds is already supported by sufficiently strong evidence. Thus, the variety of aquatic “noses,” defined as the organs that analyse odors in the external fluid medium (Atema, 2012), could be reasonably reinterpreted as aquatic “tongues.” As a striking example, the nudibranchs' “rhinophores” (the name of which means nose-bearing) that protrude into the water above the dorsal surface, could be accordingly renamed as “glossophores” (tongue-bearing). Conversely the nudibranchs' “oral tentacles” constantly touching the substrate and sensing liposoluble odorant molecules, could be regarded as the true aquatic noses (Figure 2). Similarly, the mouthpart chemosensors of crustaceans, which are used to assess food palatability (Derby and Sorensen, 2008), must be able to detect the insoluble odorant molecules too. Furthermore, although fish “nostrils” that have no connection with the mouth are used to detect waterborne molecules, many fish repeatedly take food into their oral cavity and then reject it, before swallowing or refusing it. According to our perspective, this behavior is due to the crucial need for “smelling” by contact substances that cannot be perceived at a distance. Otherwise, how could those aquatic predators avoid getting poisoned by the small liposoluble molecules contained by many benthic organisms? How can the producer organism defend itself?

**Figure 2:**
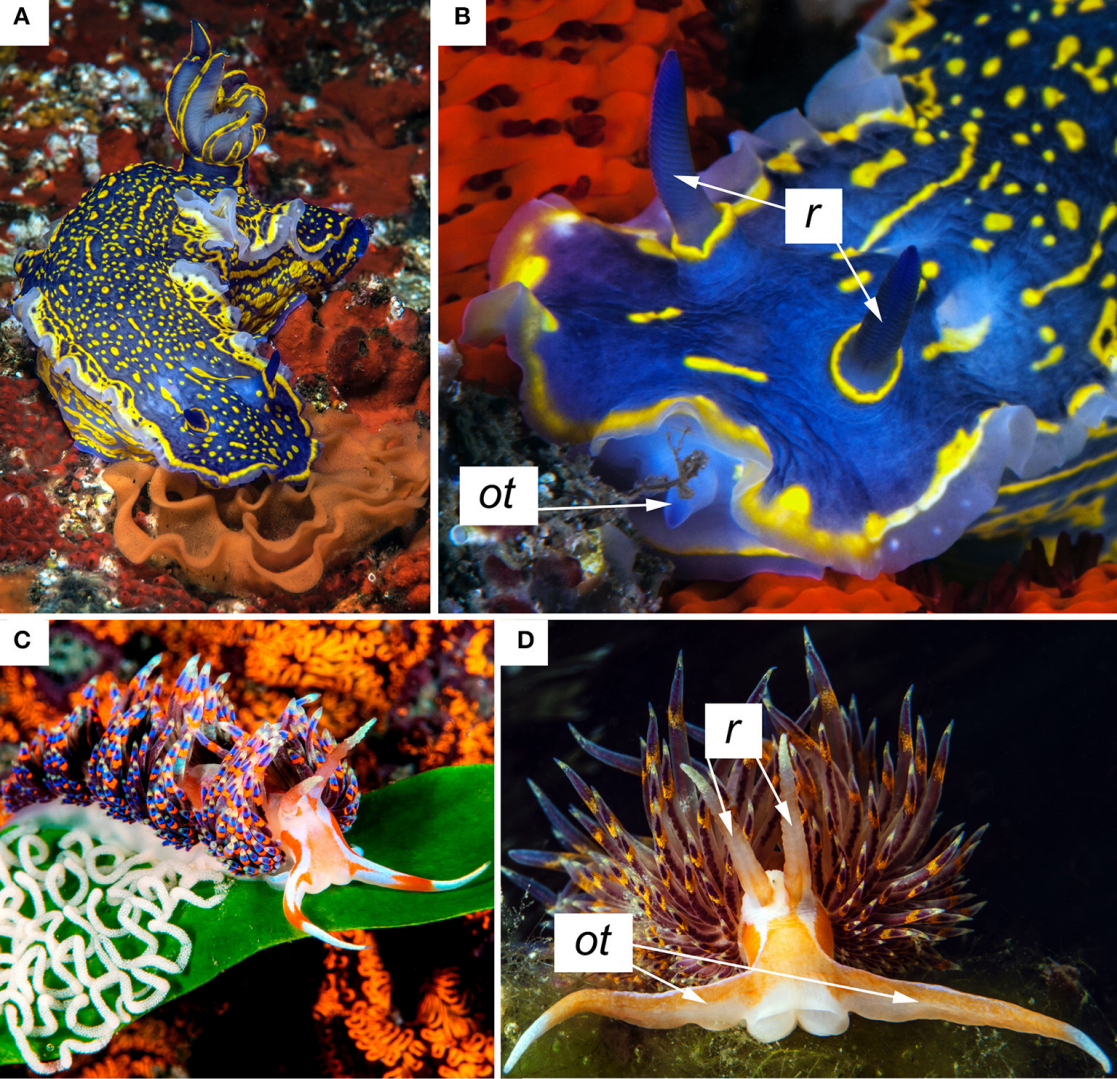
**Rhinophores (*r*) and oral tentacles (*ot*) in the nudibranchs *Felimare picta* (A,B), and *Godiva quadricolor* (C,D)**. Photos are courtesy of G. Villani.

In summary, our perspective rejects the widespread misconception that the aquatic sense of smell can be mediated only by waterborne signaling molecules. In fact, ORs-mediated aquatic chemoreception of biomolecules that combine high volatility in air and insolubility (or very low solubility) occurs, either by direct contact with the emitter, or by short-range sensing traces adherent to the substrate. On the other hand, reception of waterborne but non volatile compounds responsible for salty, sweet, bitter, sour, and umami taste perceptions, are mediated by typical TRs both at sea and on land. Thus, for signals of this kind, a “reversal of senses” in their spatial range occurs in the different environments (Figure 1).

According to this synchronic view, the terrestrial sense of smell thus derives, to a major extent, from the ORs-mediated ability of aquatic organisms to detect small hydrophobic molecules by contact, but with the expansion of its spatial range on land. Overall, our perspective suggests that during the transition from aquatic to terrestrial life, when the intrinsic “airborne” character of pre-existing water insoluble molecules appeared with their first exposure to air, the pre-existing aquatic ORs acting in contact-communication had a pre-adaptive value, predisposing the lineage to evolve remote forms of communication and all subsequent physiological, behavioral and anatomical adaptations that allow extant land plants to reduce herbivory, flowers to attract pollinators, female insects to attract the males, and humans to exploit a large variety of flavorings, drugs, poisons and perfumes.

## Conflict of interest statement

The authors declare that the research was conducted in the absence of any commercial or financial relationships that could be construed as a potential conflict of interest.
